# National Cohort Study of Long-Term Exposure to PM_2.5_ Components and Mortality in Medicare American Older Adults

**DOI:** 10.1021/acs.est.2c07064

**Published:** 2023-04-19

**Authors:** Hua Hao, Yifan Wang, Qiao Zhu, Haisu Zhang, Andrew Rosenberg, Joel Schwartz, Heresh Amini, Aaron van Donkelaar, Randall Martin, Pengfei Liu, Rodney Weber, Armistead Russel, Maayan Yitshak-sade, Howard Chang, Liuhua Shi

**Affiliations:** † Gangarosa Department of Environmental Health, Rollins School of Public Health, Emory University, Atlanta, Georgia 30322, United States; ‡ Department of Environmental Health, Harvard T.H. Chan School of Public Health, Boston, Massachusetts 02115, United States; § Department of Epidemiology, Harvard T.H. Chan School of Public Health, Boston, Massachusetts 02115, United States; ∥ Section of Environmental Health, Department of Public Health, University of Copenhagen, Copenhagen 1353, Denmark; ⊥ Department of Energy, Environmental & Chemical Engineering, Washington University at St. Louis, St. Louis, Missouri 63130, United States; # School of Earth and Atmospheric Sciences, Georgia Institute of Technology, Atlanta, Georgia 30318, United States; ¶ Department of Environmental Medicine and Public Health, Icahn School of Medicine at Mount Sinai, New York, New York 10029, United States; ∇ Department of Biostatistics and Bioinformatics, Rollins School of Public Health, Emory University, Atlanta, Georgia 30322, United States

**Keywords:** survival analysis, all-cause mortality, air pollution, PM_2.5_ components

## Abstract

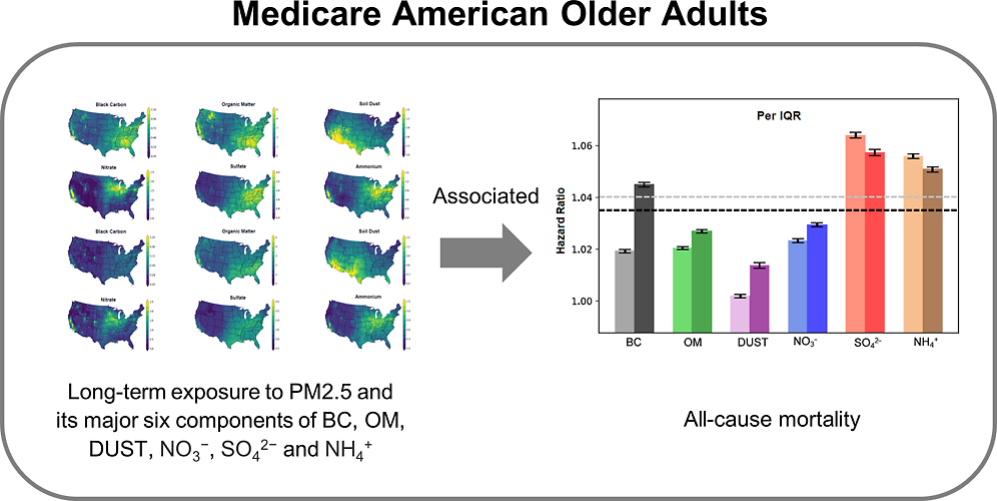

There is increasing evidence linking long-term fine particulate matter (PM_2.5_) exposure to negative health effects. However, the relative influence of each component of PM_2.5_ on health risk is poorly understood. In a cohort study in the contiguous United States between 2000 and 2017, we examined the effect of long-term exposure to PM_2.5_ main components and all-cause mortality in older adults who had to be at least 65 years old and enrolled in Medicare. We estimated the yearly mean concentrations of six key PM_2.5_ compounds, including black carbon (BC), organic matter (OM), soil dust (DUST), nitrate (NO_3_^–^), sulfate (SO_4_^2–^), and ammonium (NH_4_^+^), using two independently sourced well-validated prediction models. We applied Cox proportional hazard models to evaluate the hazard ratios for mortality and penalized splines for assessing potential nonlinear concentration–response associations. Results suggested that increased exposure to PM_2.5_ mass and its six main constituents were significantly linked to elevated all-cause mortality. All components showed linear concentration–response relationships in the low exposure concentration ranges. Our research indicates that long-term exposure to PM_2.5_ mass and its essential compounds are strongly connected to increased mortality risk. Reductions of fossil fuel burning may yield significant air quality and public health benefit.

## Abstract 2

We explored significant relationships between PM_2.5_ key compounds and mortality among the U.S. older adults, using the largest air pollution study cohort to date and two independent high-resolution speciated air pollution data sets.

## Introduction

Air pollution is among the serious environmental threats to public health. It has been well documented that long-term exposure to fine particulate matter (particles or droplets in the air that are 2.5 μm or less in diameter [PM_2.5_]) is related to higher mortality and morbidity.^1−4^ However, prior research has mainly targeted on the health consequences of PM_2.5_ mass concentrations, and the evidence of component-specific effects remains scarce.^5,6^ The main compositions of PM_2.5_ are complex, and a better awareness of compound-specific health impacts of PM_2.5_ could help guide pollution control policies by targeting more particular sources or compounds.

Toxicological and epidemiological studies suggest certain components in PM_2.5_ could have a major impact on the documented adverse health effects on humans. Animal experiments show that black carbon (BC) and sulfate (SO_4_^2–^) could harm the cardiovascular system acutely and chronically.^7,8^ Organic PM_2.5_ compounds including elemental carbon (EC) and organic carbon (OC) may have negative effects on the respiratory and immune systems.^9^ In addition, recent studies suggest that the synergistic effect of ammonium sulfate and the existence of ultrafine particles could increase the accumulation of peptides that influence the development of neurodegenerative diseases.^10^ Moreover, PM_2.5_ components of transition metals have been linked to adverse health impacts. Ostro et al. reported copper to be linked to increased ischemic heart disease mortality.^6^ Bell et al. suggested a statistically significant relationship between short-term variations in vanadium and nickel concentrations and a higher risk of cardiovascular and respiratory hospitalization.^11^ According to prior epidemiological research, short-term exposure to PM_2.5_ compounds (e.g., EC, OC, and SO_4_^2–^) may be associated with cardiovascular and respiratory outcomes.^12−14^ However, the long-term effects of exposure to PM_2.5_ compounds are still unclear.

Due to a lack of valid measurements of speciated chemical composition, it has been difficult to assess the health impacts of PM_2.5_ components. This question cannot be addressed using monitor measurements alone; particularly, high-resolution PM_2.5_ component estimations over a long period of time are required, necessitating modeling with restraints on ground-based measurement.

To overcome these gaps in knowledge, we performed a nationwide population-based cohort study (2000–2017) to examine the relationships between long-term PM_2.5_ key component exposure and all-cause mortality among the U.S. Medicare population, using two independently sourced, speciated air pollution data sets. Our study aims to find the main PM_2.5_ constituents causing the increase in mortality so that legislation can be developed to control the air quality at specific sources.

## Materials and Methods

### Study Population

We utilized the Medicare denominator file from the Centers for Medicare and Medicaid Services (CMS), a publicly available privacy-protected national database. Demographic information on sex, age, race, residential ZIP code, Medicaid eligibility (a low socioeconomic status indicator), and date of death were obtained from the denominator file for each Medicare beneficiary annually, and each beneficiary was assigned a unique ID to enable tracking over time. We created an open cohort containing all Medicare beneficiaries who were 65 years or older and residing in the contiguous United States between 2000 and 2017, and all-cause mortality was considered the outcome of interest. The CMS and Emory’s Institutional Review Board (IRB) have both given their approval for this study (#STUDY00000316 and #RSCH-2020-55733, respectively). The Medicare data set was managed and processed in a secure high-performance computing (HPC) cluster environment, compliant with the Health Insurance Portability and Accountability Act (HIPAA), at Emory Rollins School.

### Exposure Assessment

We assessed two high-resolution, speciated PM_2.5_ data sets from two independent sources for the contiguous United States from 2000 to 2017. The first set of yearly mean predictions for PM_2.5_ mass and six key components (Exposure I) was assessed at a resolution of 1 km^2^ by van Donkelaar et al. (2019).^15^ The monthly mean PM_2.5_ total mass concentrations were calculated using satellite retrievals of aerosol optical depth and a chemical transport model (CTM) and then statistically integrated with 3364 ground-based PM_2.5_ observations using geographically weighted regression. The six PM_2.5_ components were then estimated by breaking down the total mass of PM_2.5_ into specific chemical components based on CTM output and further calibrated using data from 829 unique ground-based compositional monitoring sites (402 to 821 sites depending on the chemical component). Compared with ground measurements, the predictions showed good long-term spatial agreement with cross-validated *R*^2^ values of 0.59 for BC, 0.57 for organic matter (OM), 0.60 for soil dust (DUST), 0.86 for nitrate (NO_3_^–^), 0.96 for SO_4_^2–^, and 0.90 for ammonium (NH_4_^+^).

The second set of yearly mean concentrations for key PM_2.5_ components (Exposure II) was predicted using super-learning and ensemble weighted-averaging of machine learning (ML) models, with spatial resolutions of 50 m in urban areas and 1 km^2^ in non-urban areas.^16^ Specifically, PM_2.5_ component measurements were gathered from 987 monitoring sites, and the model also took into account hundreds of additional predictors, such as traffic counts, satellite observations, CTM simulations, and meteorological variables. Six ML models in non-urban areas and three MLs in urban areas were used to forecast each component, then ensemble weighted-averaging models and multiple super-learners were applied to combine the estimates. This approach produced outstanding model performance, with a test set cross-validated *R*^2^ ranging from 0.86 to 0.96. Besides the six PM_2.5_ components, we previously estimated PM_2.5_ mass concentrations over the contiguous U.S. using an ensemble model that included hundreds of predictors and several machine learners, with a cross-validated *R*^2^ of 0.89 for annual predictions.^17^ Many other epidemiological studies have made extensive use of this PM_2.5_ mass data set.^2,18^

For each year in the study period, we calculated two sets of yearly mean concentrations for PM_2.5_ total mass and each chemical component for each ZIP code and assigned exposure values depending on the residential ZIP code of each study participant. Each subject’s residential ZIP code was updated annually, allowing us to track annual residential mobility.

### Covariates

In addition to the individual-level demographic and Medicaid eligibility data, we also included various geographic and area-level covariates in our analysis. Data included county-level behavioral risk factors (mean body mass index and smoking prevalence), county-level health care capacity variables (number of active medical doctors and hospitals), ZIP-code level Socioeconomic Status (SES) variables (percent of population with less than high school education, median household income, percent of Black population, percent of population living below the poverty line, population density, and percent of population living in rented houses or apartments), gridded meteorological variables (yearly average relative humidity and temperature), and an indicator variable for geographical region from the Behavioral Risk Factor Surveillance System (BRFSS), American Community Survey (ACS), U.S. Census Bureau, and the North American Land Data Assimilation System (NLDAS) databases, respectively. Unless otherwise stated, all covariates were incorporated into the model as linear terms. Further details on all covariates have been previously described.^2^

### Statistical Analysis

Using single-component Cox proportional hazard models, which only included one PM_2.5_ constituent at a time, we estimated the associations between each of the six chemical constituents of PM_2.5_ and all-cause mortality in older adults between 2000 and 2017. We also stratified all models by age at entry, race, sex, Medicaid eligibility (a low SES indicator), and further adjusted for area-level covariates mentioned above (e.g., behavioral risk factors, healthcare capacity, SES, and meteorological variables). To account for potential residual temporal and spatial variations, indicators of calendar year and geographic region were also incorporated into the model. All models accounted for residual autocorrelation within ZIP codes using a generalized estimating equation (GEE)^19^ to obtain robust standard errors and 95% confidence intervals (CIs). All findings are shown as hazard ratios (HRs) with 95% CIs per interquartile range (IQR) increase and per 1 μg/m^3^ increase in the mean yearly concentration of each PM_2.5_ constituent.

For each component of PM_2.5_, we fitted penalized spline models while accounting for the same covariates across models to examine any nonlinearity between the components and all-cause mortality. We introduced a penalized spline term for each relevant chemical compound in order to characterize the concentration–response (*C*–*R*) connections between each compound and mortality.

To assess the reliability of our key conclusions, we performed several sensitivity analyses. First, we fitted two sets of multi-component models by considering the potential collinearities between BC and OM and between SO_4_^2–^ and NH_4_^+^ (Figure S1). The two multi-component models were specified by (1) including BC, DUST, SO_4_^2–^, and NO_3_^–^ simultaneously (model 2), and (2) including OM, DUST, SO_4_^2–^, and NO_3_^–^ simultaneously (model 3). Second, we used an alternative exposure window with a 1 year lag period to evaluate the potential lagged effect of each compound on mortality. Mortality events were connected to exposures in the previous calendar year. Additionally, to account for potential measurement error caused by residential mobility, we performed a “non-movers” cohort analysis by restricting the analysis to individuals who remained in the same ZIP code throughout the follow-up period.

To investigate the effect modification of gender on the connections between PM_2.5_ mass and the six chemical compounds’ exposure and mortality, we stratified the data into two subgroups (male versus female), with separate regression models fit for each stratum.

R software, version 4.0.2, was employed for statistical analyses, and calculations for the analyses were performed on the Rollins HPC Cluster at Emory University.

## Results

Table 1 presents descriptive statistics for our study population from 2000 through 2017. The cohort includes approximately 73.4 million participants, of which 44.0% were male, 99.4% were between the ages of 65 and 74 at the time of enrollment, 84.1% were white, and 9.8% were eligible for Medicaid. There were 29 million deaths among the cohort (39.6%), with approximately 669.6-million person-years of follow-up and a median follow-up of 8 years. Table S1 provides additional demographic information.

**Table 1:** Descriptive Statistics for the Study Population and Ambient Air Pollution Concentrations

variables	number or mean ± SD	% or IQR
death	29,030,406	39.57%
number of total populations	73,369,159	100%
total person-years	669,680,669	100%
median follow-up years	8	
Age at Entry (Years)		
65–74	72,935,522	99.41%
≥75	433,637	0.59%
Gender		
male	32,277,229	43.99%
female	41,091,930	56.01%
Race		
white	61,708,495	84.11%
black	6,046,702	8.24%
other	5,613,962	7.65%
Medicaid Eligibility		
dual-eligible	7,191,127	9.80%
non-dual eligible	66,178,032	90.20%
Air Pollutant Concentrations (μg/m^3^)—Exposure I^a^		
PM_2.5_ mass	9.30 ± 2.90	3.68
black carbon	0.77 ± 0.33	0.33
organic matter	3.31 ± 1.11	1.19
soil dust	0.63 ± 0.39	0.38
nitrate	1.18 ± 0.80	1.06
sulfate	2.23 ± 1.19	1.73
ammonium	0.89 ± 0.54	0.83
Air Pollutant Concentrations (μg/m^3^)—Exposure II^a^		
PM_2.5_ mass	10.03 ± 3.12	4.07
black carbon	0.57 ± 0.25	0.29
organic matter	3.03 ± 1.00	1.19
soil dust	0.68 ± 0.31	0.34
nitrate	1.12 ± 0.62	0.92
sulfate	2.19 ± 1.17	2.08
ammonium	0.87 ± 0.49	0.82

a Exposure I pollutants were derived from van Donkelaar et al. (2019), and Exposure II pollutants were derived from Amini et al. (2022). SD indicates standard deviation; IQR indicates interquartile range.

Using Exposure I data, for the period 2000–2017, the mean PM_2.5_ mass concentration was 9.30 μg/m^3^ (IQR of 3.68 μg/m^3^). The average concentration of each PM_2.5_ major component was 0.77 μg/m^3^ (BC), 3.31 μg/m^3^ (OM), 0.63 μg/m^3^ (DUST), 1.18 μg/m^3^ (NO_3_^–^), 2.23 μg/m^3^ (SO_4_^2–^), and 0.89 μg/m^3^ (NH_4_^+^); the corresponding IQRs were 0.37, 1.18, 0.32, 1.07, 1.73, and 0.80 μg/m^3^, respectively. With the exception of BC, where the Exposure II data indicated slightly lower levels than Exposure I, all other component distributions were comparable between the two exposure data.

**Figure 1:**
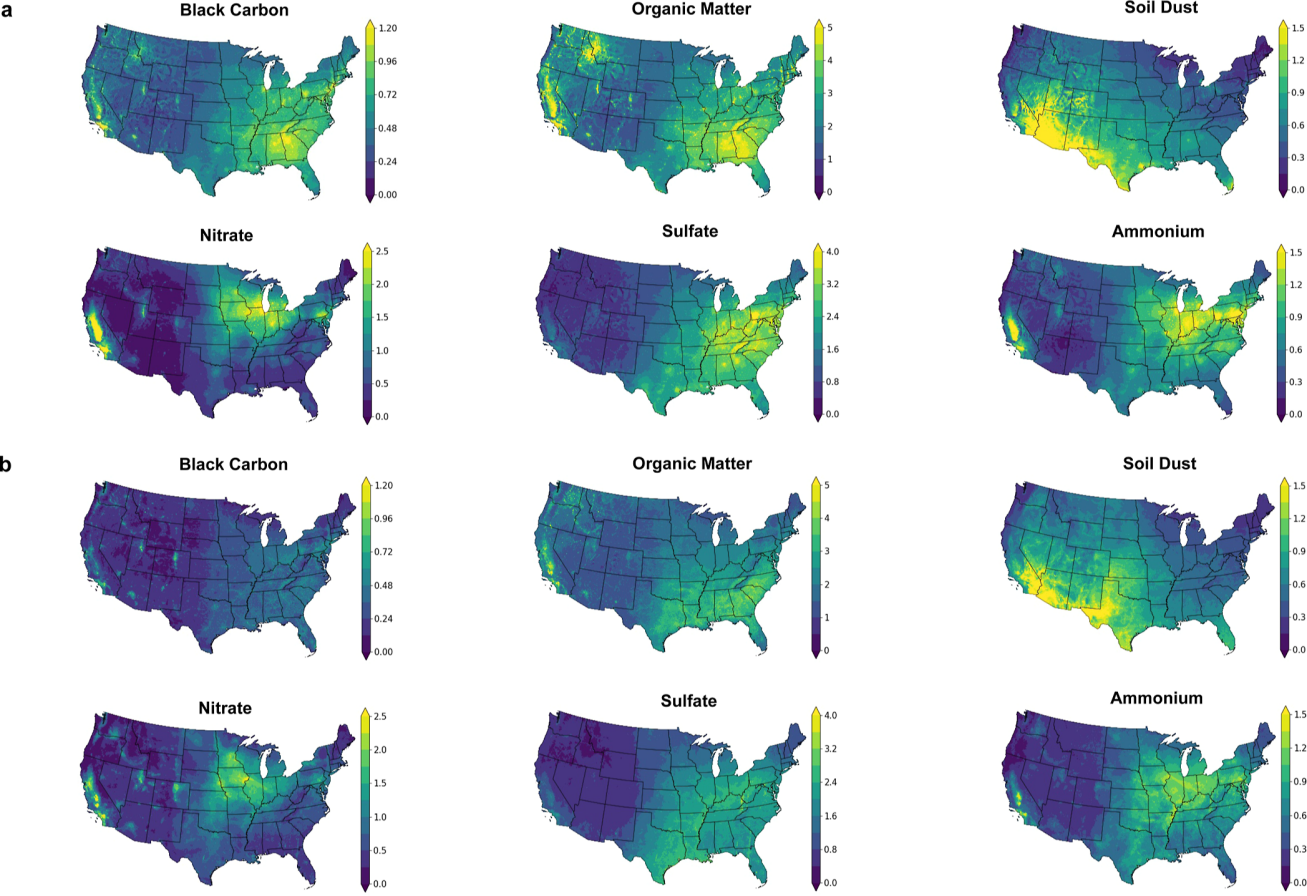
Average concentrations of PM_2.5_ major components (μg/m^3^) over the contiguous United States from 2000 to 2017 as calculated by Exposure I (van Donkelaar et al., 2019) (a) and Exposure II (Amini et al., 2022) (b).

The spatial distributions of each chemical component were generally consistent between the two data sets (Figure 1). Figure S1 shows the correlation matrix among PM_2.5_ mass and the six major compounds at the cohort level. PM_2.5_ mass was highly correlated with BC, OM, NO_3_^–^, SO_4_^2–^, and NH_4_^+^ in Exposure I (*r* values range from 0.66 to 0.81) and highly correlated with NH_4_^+^ (*r* = 0.81) and SO_4_^2–^ (*r* = 0.74) in Exposure II. Strong correlations were also indicated by BC and OM (*r* = 0.79 and 0.69) and SO_4_^2–^ and NH_4_^+^ (*r* = 0.80 and 0.85) in both exposure sets. Figure S2 shows the average chemical composition of the six components of PM_2.5_ total mass in each exposure data set. Between 2000 and 2017, we observed similar proportions between chemical components across exposure data sets, with OM and SO_4_^2–^ accounting for the largest proportions of total PM_2.5_ mass concentrations.

**Figure 2:**
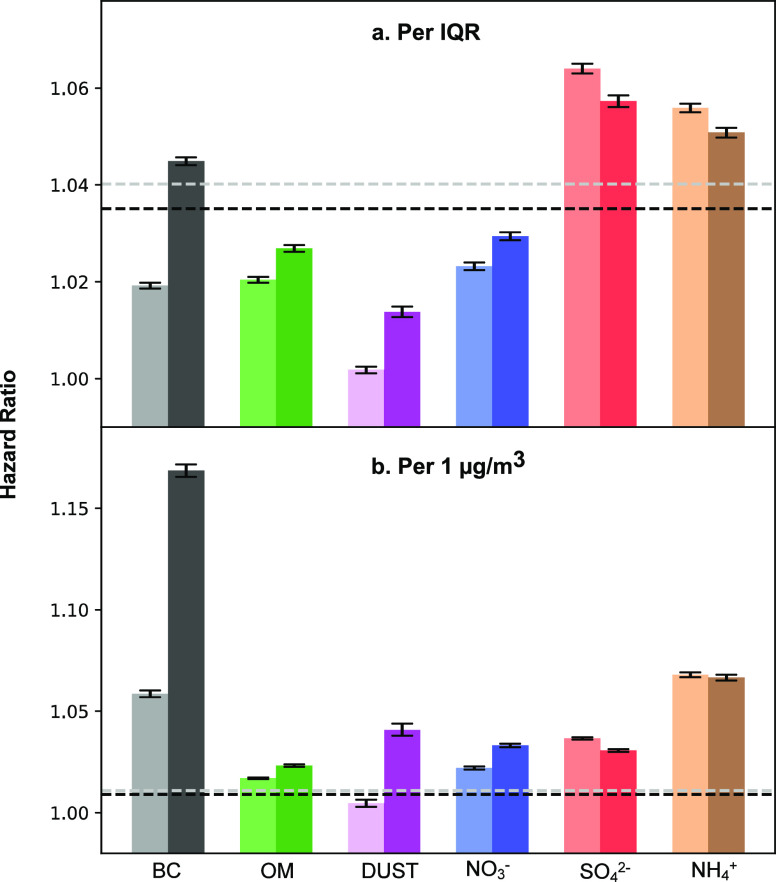
HRs of mortality linked with per IQR (a) or per 1 μg/m^3^ (b) increase in annual mean concentration of each PM_2.5_ major component, respectively, including BC, OM, soil dust (DUST), nitrate (NO_3_^–^), sulfate (SO_4_^2–^), and ammonium (NH_4_^+^). The dotted lines represent PM_2.5_ mass results. The error bars represent the 95% confidence intervals, while the calculated HRs were determined from single-component models. Air pollutants derived from two exposure models are distinguished using light and dark colors, with the light color denoting Exposure I data and the dark color denoting Exposure II data. Table S3 provides the corresponding hazard ratio values (model 1).

Using Exposure I data, the single-component models indicate a significant positive relationship between long-term exposure to PM_2.5_ total mass and its six major compounds and all-cause mortality (Figure 2). Per IQR increase in the concentrations, the HR of mortality were 1.040 (95% confidence interval [CI]: 1.039, 1.041) for PM_2.5_ mass, 1.019 (CI: 1.019, 1.020) for BC, 1.020 (CI: 1.020, 1.021) for OM, 1.002 (CI: 1.001, 1.002) for DUST, 1.064 (CI: 1.063, 1.065) for SO_4_^2–^, 1.023 (CI: 1.022, 1.024) for NO_3_^–^, and 1.056 (CI: 1.055, 1.057) for NH_4_^+^ (Table S3). Using Exposure II data, single-component models yielded larger effect estimates for BC and DUST and similar results for all other chemical components. Per 1 μg/m^3^ increase, the highest estimated risk of mortality was observed for BC (Exposure I: HR: 1.059 [CI: 1.057, 1.060]; Exposure II: HR: 1.169 [CI: 1.165, 1.172] and NH_4_^+^ (Exposure I: HR: 1.068 [CI: 1.067, 1.069]; Exposure II: HR: 1.067 [CI: 1.065, 1.068] (Table S3).

**Figure 3:**
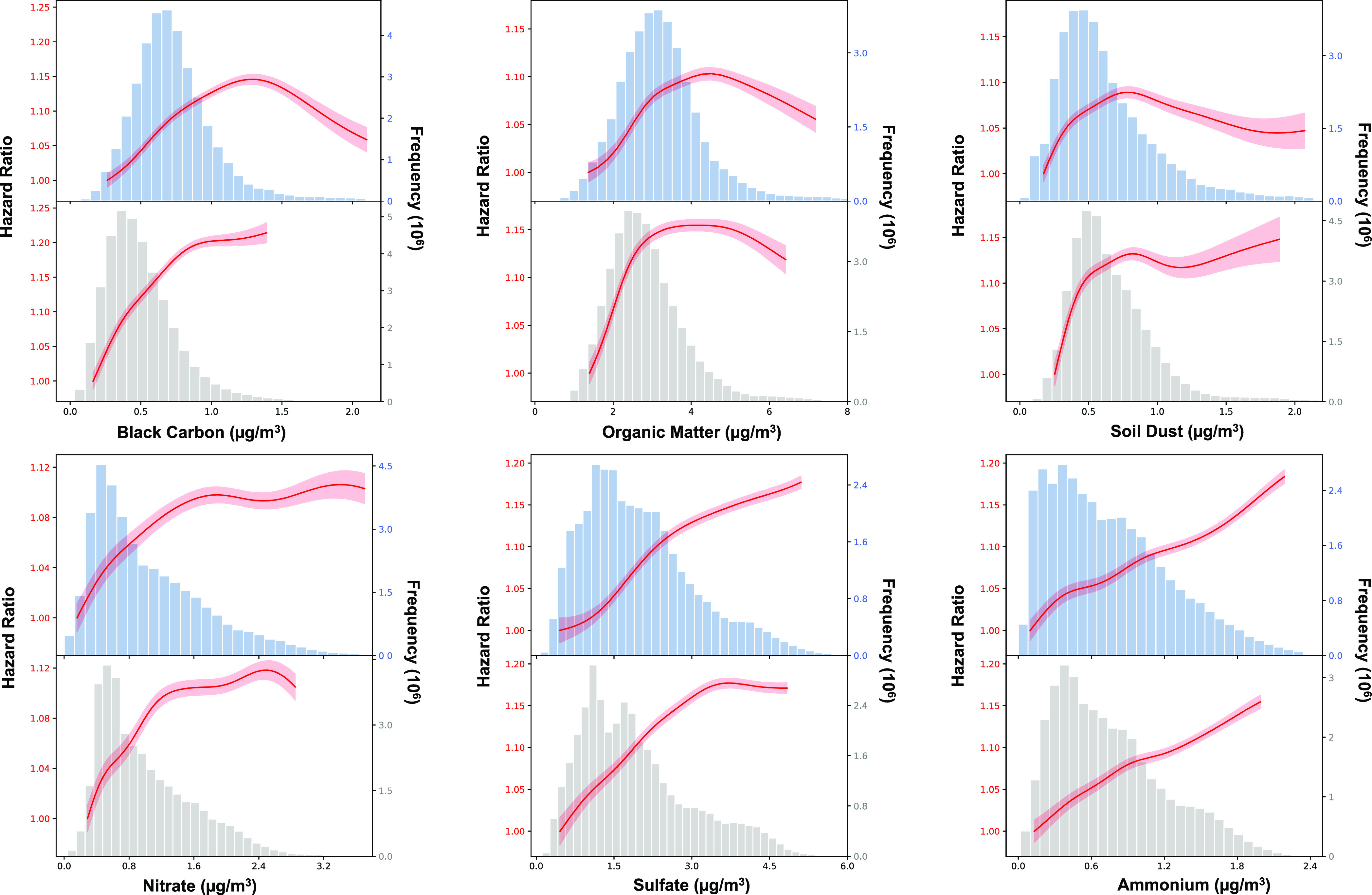
Concentration–response curves, derived from the single-component models, are displayed for the concentration ranges between the 1st to 99th percentiles of the pollutants, i.e., with 2% of the extreme values that are poorly constrained removed. For each component, the top panel shows the Exposure I result, and the bottom panel shows the Exposure II result.

Figure 3 shows the calculated *C*–*R* relationship for each of the six chemical components. The shape of the *C*–*R* curve revealed an approximately linear relationship for BC (Exposure II), NO_3_^–^, SO_4_^2–^, and NH_4_^+^, with no clear indication of a threshold for all-cause mortality. The *C*–*R* relationship for BC (Exposure I) tends to be linear across the majority of data, though not at the extremes of the exposure distribution (>1.30 μg/m^3^). Near-linear relationships were discovered for OM (<4 μg/m^3^) and DUST (<1 μg/m^3^) in the mid-range of exposure distributions, though leveled off at higher concentrations.

Our findings were robust to several sensitivity analyses. First, under multi-component models, we found similar results for most PM_2.5_ components, excluding DUST, which yielded a weaker, non-significant association after adjusting for other PM_2.5_ components using Exposure I data (Table S3). Second, we observed consistency in our results after specifying a 1 year lag between annual exposure for each chemical component and mortality (Table S4). Lastly, we found minimal bias due to residential mobility in our analysis of the “non-movers” cohort (Table S5).

Table S6 shows subgroup-specific results stratified by gender. In both gender strata, we discovered that the total mass of PM_2.5_ and its six main compounds remained significantly positively associated with all-cause death. Using the estimations from Exposures I and II, comparable patterns were discovered in most cases. Effect estimates for OM and SO_4_^2–^ were higher among male subjects in both exposure data. For NO_3_^–^ and NH_4_^+^, effect estimates were higher among female subjects in both exposure data.

## Discussion

We used two independently sourced data sets of high-resolution speciated PM_2.5_ data to estimate the long-term effects of exposure to PM_2.5_ chemical components on all-cause mortality in a nationwide, population-based cohort of older adults. We found that long-term exposure to PM_2.5_ total mass and its key chemical constituents was significantly associated with an increased risk of all-cause mortality among U.S. older adults. Specifically, among each of the six key compounds of PM_2.5_ studied, we found the strongest associations for SO_4_^2–^, NH_4_^+^, and BC, while DUST had a relatively weaker impact, which is consistent with literature that DUST is typically not the predominant factor.^20^ Adjusting for other pollutants in multi-component models only modestly changed the effect estimates except for DUST, indicating that the other components did not confound the observed associations.

Overall, SO_4_^2–^ and NH_4_^+^ had the largest estimated effect on mortality per IQR change in exposure among all six PM_2.5_ components, followed by NO_3_^–^. NH_4_^+^ is chemically associated with SO_4_^2–^ and NO_3_^–^, and its effect estimates are as expected in between. SO_4_^2–^, NH_4_^+^, and NO_3_^–^ are three predominant secondary inorganic aerosols in PM_2.5_ and are significantly associated with increased mortality in our study. They are typically present as ammonium sulfate ((NH_4_)_2_SO_4_), ammonium bisulfate (NH_4_HSO_4_), and ammonium nitrate (NH_4_NO_3_). A recently published panel study demonstrated that the hypothalamic–pituitary–adrenal axis can be activated by exposure to SO_4_^2–^, NH_4_^+^, and NO_3_^–^, which may affect the cardiovascular system.^21^ Previous large-scale epidemiology studies also suggested that secondary inorganic aerosols were connected to all-cause, cardiovascular disease, and cardiopulmonary mortality in Denmark,^22^ China,^23^ and U.S.^24^ Sulfur dioxide (SO_4_^2–^ precursor) has natural sources (e.g., oceans and volcanic emissions) and anthropogenic emissions primarily from fossil fuel combustion. SO_4_^2–^ can alter bronchial mucociliary transport in humans,^25^ change the alveolar macrophage function,^26^ and influence aortic contraction.^7^ Additionally, SO_4_^2–^ provides an acidic environment in the atmosphere and facilitates the solubility and bioavailability of trace metals in fine particulate matter,^27^ which in turn causes reactive oxygen species (ROS) to be produced. ROS cause oxidative stress, inflammation, and genotoxicity, which are conditions that damage cellular physiological processes.^28^

Nitrogen dioxide, an NO_3_^–^ precursor, is primarily derived from fossil fuel combustion. Previous studies suggest that exposure to NO_3_^–^ is associated with a circulatory biomarker of tumor necrosis factor alpha (TNF-α).^29^ An elevated level of TNF-α may play a role in vascular dysfunction of the cardiovascular system, atherosclerosis formation and progression, and negative cardiac remodeling after myocardial infarction and heart failure.^30^ In addition, animal studies have reported that exposure to NO_3_^–^ may result in lung inflammatory cell infiltration, alveoli collapse, and thickening of the small airway wall.^31^ We also discovered a strong link between NH_4_^+^ and mortality, and the strength of the observed association was consistent with previous studies^23,32^ that the effect of NH_4_^+^ ranked top among the contributing factors. However, it is unclear whether the observed associations are due to their intrinsic toxicity or because they are associated with other combustion-emitted culprit pollutants.

BC was observed to have the largest effect estimates on mortality per 1 μg/m^3^ change in exposure, although a 1 μg/m^3^ increase is a much larger relative increase for BC than other components studied (e.g., OM and sulfate) as shown in Table 1. BC emissions mainly come from incomplete combustion of biomass and fossil fuel and traffic emission.^33^ We observed larger effect sizes using Exposure II data compared to Exposure I (Figure 2). This discrepancy in the effect size for BC may be explained, in part, by the discrepancy in BC concentrations of the two exposure sets that relied on monitored data from different sources (i.e., thermal method vs optical method). There is some epidemiological evidence linking BC with mortality. BC has been considered as one of the specific markers for traffic-related air pollution,^34^ and traffic-related air pollution has already been linked with lung cancer^35^ and cardiorespiratory deaths^36^ in many previous epidemiology studies. A World Health Organization (WHO) report summarized associations between BC with all-cause, cardiopulmonary, and cardiovascular mortality.^37^ A previous large cohort study from the American Cancer Society also reported that coal combustion and fossil fuel combustion PM_2.5_ were strongly and robustly associated with ischemic heart disease mortality.^38^ The underlying biological mechanism of association between BC exposure and mortality can be characterized by the cardiopulmonary system. It is generally understood that exposure may induce oxidative DNA damage, increase levels of inflammatory mediators and oxidative stress, in addition to blood–brain barrier disruption, which can facilitate cardiovascular diseases such as hypertension, atherosclerosis, and stroke.^8,39−41^ One in vitro study^42^ suggests that BC could directly affect vascular endothelium triggering cytotoxic injury, inflammatory responses, and cell growth suppression.

Another chemical component linked to premature mortality is OM, which constituted a large fraction of PM_2.5_ mass in both exposure data sets in the present study (Figure S2). OM can be released directly from biogenic sources and combustion emissions, or secondarily formed through oxidation of volatile organic compounds (VOCs) and reactions that transform VOCs into low-vapor-pressure compounds that can condense on existing particles.^43^ The latter secondary formation process for OM can change the toxicity of original particles. For instance, polycyclic aromatic hydrocarbons (PAHs) and polychlorinated biphenyls (PCBs) are highly toxic OM species, which are always found in OC.^44^ PAHs and PCBs are known to cause a variety of adverse health effects in the reproductive system, immune system, and nervous system.^45,46^ Additionally, OM can induce adverse cardiovascular effects via negative changes in blood pressure, heart rate variability, and worsening biomarker levels reflecting inflammation, hemostasis, and oxidative stress.^29,47,48^

We observed linear relationships with BC (Exposure II), SO_4_^2–^, and NH_4_^+^, with no indication of a threshold for either outcome, and the previously published studies in Southeastern U.S. and China also reported similar linear association for BC (Exposure II), NO_3_^–^, and SO_4_^2–^.^20,23^ We observed nonlinear “bell-shaped” *C*–*R* relationships between exposure to BC (Exposure I), OM, and DUST and mortality, indicating that the relationships are increasing steeply at low to moderate exposure levels and leveled off at high exposure levels. There have been many theories proposed to explain the reasons causing a nonlinear *C*–*R* relationship, including preferential avoidance based on symptoms, decreased inhalation, and errors in estimating pollution exposure levels at elevated concentrations.^49^ Furthermore, different *C*–*R* curves might result from the variations in population distributions across different components. Various study areas, populations, exposure time windows, and other factors could account for the disparity in *C*–*R* associations between our study and other studies.^50^

According to our knowledge, this is the first nationwide cohort research to explore relationships between major chemical components of PM_2.5_ and all-cause mortality in the U.S. In Europe, there are studies investigating the relationship between long-term exposure to PM_2.5_ metal constituents and mortality, but the results are inconsistent. Chen et al.^32^ reported in single pollutant models that all eight metals (Fe, Cu, K, S, Ni, Si, Zn, and V) had statistically significant associations with natural-cause mortality with HRs ranging from 1.05 to 1.27. But Wang et al.^51^ did not report any significant relationships between cardiovascular deaths and those eight metals. Our component-specific study provides novel insight into the long-term effects of exposure to PM_2.5_ focusing on the individual impact of the main chemical compounds of PM_2.5_ total mass on mortality. Moreover, the use of two independently sourced, high-resolution exposure data sets allowed us to assess the validity of our results under different exposure assessment models, increasing our confidence in observed associations. We treated the two exposure data sets equally in this study, since the models from which the two exposures were derived were based on different algorithms, and each of the methods has its respective pros and cons. Even though we used both exposure data sets to explore the relationship between PM_2.5_ constituents and all-cause mortality, consistent results of single- and multi-constituent models were still observed, and this can strongly demonstrate the robustness of this association. Additionally, the large national cohort provides sufficient statistical power to capture complicated spatiotemporal patterns and variations in PM_2.5_ composition and mortality risk, which may otherwise bias results in small samples.

Our study has several limitations. First, although the exposure assessment model achieved a high performance, the use of projected surface pollutant concentrations may still result in measurement error, despite showing strong model performance. Second, although our statistical models were adjusted for many potential confounders, we acknowledge that unmeasured individual-level risk factors (e.g., smoking, drug use, alcohol use) linked to premature mortality may have biased risk estimates.^52,53^ However, the individual level variables were less likely to be confounders since our exposure was assigned on a ZIP code level. Furthermore, multicollinearity is likely due to correlations among the chemical components of PM_2.5._ as such computationally scalable speciation techniques are needed to address this issue in large-scale epidemiological studies. Although the six major chemical components explored in this study accounted for most of PM_2.5_ total mass, we cannot rule out the possibility that other unexamined and potentially correlated components confer risk. Lastly, studies of PM_2.5_ components may be hard to interpret because the identification of individual emission sources of PM_2.5_ chemical components, particularly the extent of spatial variability in local sources, is a considerable challenge. Future studies assessing source-specific effects of PM_2.5_ will be important as they can be readily translatable into more targeted and effective air pollution control policies.

In conclusion, our study demonstrates that long-term exposure to PM_2.5_ mass and its major components was related to an elevated risk of all-cause mortality among U.S. older adults. Reductions of PM_2.5_ emission sources, such as fossil fuel burning (especially for BC exposure in high level areas), and traffic and power plants emissions, can lead to substantial public health benefits.

## Supporting Information Available

Details regarding area-level covariates; sensitivity analysis; correlations among PM_2.5_ and its six major components (BC, OM, soil dust, nitrate, sulfate, and ammonium); and averaged chemical composition of PM_2.5_ for two air pollution data sets (PDF)

## Notes

The secure cluster environment at the Emory Rollins School was utilized to store and analyze the Medicare data set in accordance with HIPAA regulations. The health data sets used for our research cannot be shared due to the Medicare data set rule. However, the same data sets may be obtained by other investigators from CMS upon application and completion of their own Data Use Agreement. The Medicare data that supported our research conclusions are not publicly available from us and cannot be shared because of restrictions imposed by our data use agreement with the U.S. Centers for Medicare & Medicaid Services. To obtain Medicare data, non-profit and academic researchers should approach the US Centers for Medicare & Medicaid Services.
