# Supplementary material for: National Cohort Study of Long-Term Exposure to PM2.5 Components and Mortality in Medicare American Older Adults
Source: Environ Sci Technol. 2023 Apr 19;57(17):6835–43. doi: 10.1021/acs.est.2c07064 (PMC10157884; doi:10.1021/acs.est.2c07064)
Supplement: Supplementary file 1 — es2c07064_si_001.pdf [file es2c07064_si_001.pdf]

Supplementary Appendix for

A National Cohort Study of Long-term Exposure to $PM_{2.5}$ Components and Mortality in Medicare American Older Adults

Hua Hao[1], Ph.D., Yifan Wang[1], M.S., Qiao Zhu[1], Ph.D., Haisu Zhang[1], M.S., Andrew Rosenberg[1], M.P.H., Joel Schwartz[2,3], Ph.D., Heresh Amini[4], Ph.D., Aaron van Donkelaar[5], Ph.D., Randall Martin[5], Ph.D., Pengfei Liu[6], Ph.D., Rodney Weber[6], Ph.D., Armistead Russel[6], Ph.D., Maayan Yitshak-sade[7], Ph.D., Howard Chang[8], Ph.D., Liuhua Shi[1*], Sc.D.

[1]Gangarosa Department of Environmental Health, Rollins School of Public Health, Emory University, Atlanta, Georgia, 30322, USA
[2]Department of Environmental Health, Harvard T.H. Chan School of Public Health, Boston, Massachusetts, 02115, USA
[3]Department of Epidemiology, Harvard T.H. Chan School of Public Health, Boston, Massachusetts, 02115, USA
[4]Section of Environmental Health, Department of Public Health, University of Copenhagen, Copenhagen, 1353, Denmark
[5]Department of Energy, Environmental & Chemical Engineering, Washington University at St. Louis, Missouri, 60130, USA
[6]School of Earth and Atmospheric Sciences, Georgia Institute of Technology, Atlanta, Georgia, 30318, USA
[7]Department of Environmental Medicine and Public Health, Icahn School of Medicine at Mount Sinai, New York, 10029, USA
[8]Department of Biostatistics and Bioinformatics, Rollins School of Public Health, Emory University, Atlanta, Georgia, 30322, USA

Correspondence to: Liuhua Shi, Gangarosa Department of Environmental Health, Rollins School of Public Health, Emory University, Atlanta, Georgia, 30322, USA

*Email: liuhua.shi@emory.edu

This file contains 12 pages, including 6 tables and 2 figures.

Table of Contents

Table S1. Summary statistics on area-level covariates among the mortality cohort (2000-2017)........S3

Table S2. Annual $PM_{2.5}$ major components (μg/m^3) levels (minimum, maximum, mean, and percentiles) over the study period (2000-2017)........S4

Table S3. Hazard ratios of death per interquartile range (IQR) or per 1 μg/m^3 increase in $PM_{2.5}$ components from single-component and multi-component models........S5

Table S4. Hazard ratios of death per interquartile range (IQR) or per 1 μg/m^3 increase in $PM_{2.5}$ components from single-component models........S7

Table S5. Hazard ratios of death per interquartile range (IQR) or per 1 μg/m^3 increase in $PM_{2.5}$ components from single-component models, among the non-mover cohort........S9

Table S6. Hazard ratios of death per interquartile range (IQR) or per 1 μg/m^3 increase in $PM_{2.5}$ components from single-component models, among male and female subjects........S10

Figure S1. Correlation matrix among $PM_{2.5}$ mass and its six major components (black carbon, organic matter, soil dust, nitrate, sulfate, and ammonium) for two speciated air pollution datasets (a: Exposure I; b: Exposure II)........S11

Figure S2. Averaged chemical composition of $PM_{2.5}$ for two speciated air pollution datasets (a: Exposure I; b: Exposure II) from 2000 to 2017, including black carbon (BC), organic matter (OM), soil dust (DUST), nitrate (NO_3^-), sulfate (SO_4^{2-}), ammonium (NH_4^+), and others........S12

Table S1. Summary statistics on area-level covariates among the mortality cohort (2000-2017).

**Variables**	**Mortality Cohort (N=73,369,159)**	
	**Mean**	**SD**
Mean body mass index, kg/m²	27.06	1.07
Smoking prevalence, %	46.52	7.38
Black, %	10.98	17.85
Renting house or apartment, %	31.91	15.99
Population living below the poverty line, %	12.57	7.99
Not graduated from high school, %	17.11	10.13
Median household income, $1000	50.62	21.32
Number of hospitals	12.91	21.03
Number of active medical doctors	3,350.34	5,790.72
Population density, people per km²	1,295.33	3,308.28

Table S2. Annual $PM_{2.5}$ major components ($\mu g/m^3$) levels (minimum, maximum, mean, and percentiles) over the study period (2000-2017).

Pollutants	Min	0.5th	1st	25th	50th	75th	99th	99.5th	Max	Mean
$PM_{2.5}$ mass [a]	0.68	3.03	3.44	7.49	9.12	11.18	17.15	19.37	37.30	9.30
$PM_{2.5}$ mass [b]	0.01	2.85	3.28	7.94	9.81	12.01	17.87	20.19	30.92	10.03
Black carbon [a]	0.00	0.21	0.25	0.57	0.72	0.90	2.10	2.41	3.25	0.77
Black carbon [b]	0.03	0.12	0.15	0.40	0.52	0.69	1.40	1.54	2.62	0.57
Organic matter [a]	0.16	1.10	1.27	2.62	3.19	3.81	7.39	7.91	24.14	3.31
Organic matter [b]	0.66	1.25	1.37	2.35	2.85	3.54	6.44	6.90	11.02	3.03
Soil dust [a]	0.00	0.11	0.13	0.38	0.54	0.77	2.11	2.42	4.27	0.63
Soil dust [b]	0.09	0.21	0.24	0.47	0.61	0.81	1.89	2.04	3.33	0.68
Nitrate [a]	0.00	0.11	0.14	0.56	0.98	1.62	3.74	4.42	7.80	1.18
Nitrate [b]	0.04	0.21	0.26	0.61	0.97	1.53	2.85	3.23	4.92	1.12
Sulfate [a]	0.08	0.33	0.39	1.27	2.08	3.00	5.17	5.44	7.51	2.23
Sulfate [b]	0.12	0.38	0.43	1.20	1.95	3.06	4.84	5.02	6.89	2.19
Ammonium [a]	0.00	0.02	0.09	0.44	0.82	1.26	2.22	2.31	3.23	0.89
Ammonium [b]	0.02	0.08	0.11	0.45	0.80	1.23	2.01	2.09	2.81	0.87

Note: [a]Pollutants were derived from Exposure I (van Donkelaar et al., 2019); [b]Pollutants were derived from Exposure II (Amini et al., 2022). DUST was calculated using an empirical formula (2.20Al + 2.49Si + 1.63Ca + 1.94Ti + 2.42Fe) from Shiraki and Holmen (2002). Due to the lack of Al and Ti, we modified the empirical formula as (2.49×Si+1.63×Ca+2.42×Fe)×1.4 for compensation of concentration level in Exposure II, and 1.4 is the fitted slope of DUST in Exposure I and (2.49×Si+1.63×Ca+2.42×Fe) in Exposure II.

Table S3. Hazard ratios of death per interquartile range (IQR) or per 1 μg/m^3 increase in $PM_{2.5}$ components from single-component and multi-component models.

Pollutant	Model 1	Model 2	Model 3
**Per IQR increase in Exposure I pollutant***			
**Black carbon**	1.019 (1.019, 1.020)	1.004 (1.003, 1.005)	_
**Organic matter**	1.020 (1.020, 1.021)	_	1.011 (1.011, 1.012)
**Soil dust**	1.002 (1.001, 1.002)	0.999 (0.999, 1.000)	1.000 (0.999, 1.000)
**Nitrate**	1.023 (1.022, 1.024)	1.007 (1.006, 1.008)	1.002 (1.002, 1.003)
**Sulfate**	1.064 (1.063, 1.065)	1.058 (1.057, 1.059)	1.057 (1.056, 1.058)
**Ammonium**	1.056 (1.055, 1.057)	_	_
**$PM_{2.5}$ mass**	1.040 (1.039, 1.041)	_	_
**Per 1 μg/m^3 increase in Exposure I pollutant**			
**Black carbon**	1.059 (1.057, 1.060)	1.012 (1.01, 1.014)	_
**Organic matter**	1.017 (1.017, 1.017)	_	1.010 (1.009, 1.010)
**Soil dust**	1.005 (1.003, 1.006)	0.999 (0.997, 1.000)	0.999 (0.997, 1.001)
**Nitrate**	1.022 (1.021, 1.023)	1.006 (1.006, 1.007)	1.002 (1.002, 1.003)
**Sulfate**	1.037 (1.036, 1.037)	1.033 (1.033, 1.034)	1.033 (1.032, 1.033)
**Ammonium**	1.068 (1.067, 1.069)	_	_
**$PM_{2.5}$ mass**	1.011 (1.011, 1.011)	_	_
**Per IQR increase in Exposure II pollutant***			
**Black carbon**	1.045 (1.044, 1.046)	1.035 (1.034, 1.036)	_
**Organic matter**	1.027 (1.026, 1.028)	_	1.015 (1.014, 1.016)
**Soil dust**	1.014 (1.013, 1.015)	1.004 (1.003, 1.005)	1.017 (1.016, 1.018)
**Nitrate**	1.029 (1.029, 1.030)	1.002 (1.001, 1.003)	1.011 (1.010, 1.012)
**Sulfate**	1.057 (1.056, 1.058)	1.040 (1.039, 1.041)	1.042 (1.041, 1.044)
**Ammonium**	1.051 (1.050, 1.052)	_	_
**$PM_{2.5}$ mass**	1.035 (1.034, 1.036)	_	_
**Per 1 μg/m^3 increase in Exposure II pollutant**			
**Black carbon**	1.169 (1.165, 1.172)	1.130 (1.126, 1.134)	_
**Organic matter**	1.023 (1.023, 1.024)	_	1.013 (1.012, 1.013)
**Soil dust**	1.041 (1.038, 1.044)	1.011 (1.008, 1.014)	1.052 (1.048, 1.055)
**Nitrate**	1.033 (1.032, 1.034)	1.003 (1.002, 1.004)	1.013 (1.012, 1.014)
**Sulfate**	1.031 (1.030, 1.031)	1.021 (1.021, 1.022)	1.023 (1.022, 1.023)
**Ammonium**	1.067 (1.065, 1.068)	_	_
**$PM_{2.5}$ mass**	1.009 (1.008, 1.009)	_	_

Note:
Model 1: Single-component model.

Model 2: Multi-component model, including black carbon, soil dust, sulfate, and nitrate in one model simultaneously.
Model 3: Multi-component model, including organic matter, soil dust, sulfate, and nitrate in one model simultaneously.
***** Hazard ratios were calculated using the same IQRs as described in Table 1.
Exposure I pollutants were derived from van Donkelaar et al. (2019).
Exposure II pollutants were derived from Amini et al. (2022).

Table S4. Hazard ratios of death per interquartile range (IQR) or per 1 μg/m^3 increase in $PM_{2.5}$ components from single-component models.

Pollutant	Lag 0#	Lag 1#
**Per IQR increase in Exposure I pollutant***		
**Black carbon**	1.019 (1.019, 1.020)	1.019 (1.019, 1.020)
**Organic matter**	1.020 (1.020, 1.021)	1.020 (1.020, 1.021)
**Soil dust**	1.002 (1.001, 1.002)	1.001 (1.000, 1.002)
**Nitrate**	1.023 (1.022, 1.024)	1.023 (1.022, 1.024)
**Sulfate**	1.064 (1.063, 1.065)	1.065 (1.064, 1.066)
**Ammonium**	1.056 (1.055, 1.057)	1.057 (1.056, 1.058)
**$PM_{2.5}$ mass**	1.040 (1.039, 1.041)	1.040 (1.040, 1.041)
**Per 1 μg/m^3 increase in Exposure I pollutant**		
**Black carbon**	1.059 (1.057, 1.060)	1.059 (1.057, 1.060)
**Organic matter**	1.017 (1.017, 1.017)	1.017 (1.017, 1.018)
**Soil dust**	1.005 (1.003, 1.006)	1.003 (1.001, 1.005)
**Nitrate**	1.022 (1.021, 1.023)	1.022 (1.021, 1.023)
**Sulfate**	1.037 (1.036, 1.037)	1.037 (1.037, 1.038)
**Ammonium**	1.068 (1.067, 1.069)	1.069 (1.068, 1.070)
**$PM_{2.5}$ mass**	1.011 (1.011, 1.011)	1.011 (1.011, 1.011)
**Per IQR increase in Exposure II pollutant***		
**Black carbon**	1.045 (1.044, 1.046)	1.044 (1.044, 1.045)
**Organic matter**	1.027 (1.026, 1.028)	1.027 (1.026, 1.027)
**Soil dust**	1.014 (1.013, 1.015)	1.014 (1.013, 1.015)
**Nitrate**	1.029 (1.029, 1.030)	1.030 (1.029, 1.030)
**Sulfate**	1.057 (1.056, 1.058)	1.059 (1.058, 1.060)
**Ammonium**	1.051 (1.050, 1.052)	1.052 (1.051, 1.053)
**$PM_{2.5}$ mass**	1.035 (1.034, 1.036)	1.036 (1.035, 1.036)
**Per 1 μg/m^3 increase in Exposure II pollutant**		
**Black carbon**	1.169 (1.165, 1.172)	1.167 (1.164, 1.170)
**Organic matter**	1.023 (1.023, 1.024)	1.023 (1.022, 1.024)
**Soil dust**	1.041 (1.038, 1.044)	1.041 (1.038, 1.044)
**Nitrate**	1.033 (1.032, 1.034)	1.033 (1.032, 1.034)
**Sulfate**	1.031 (1.030, 1.031)	1.032 (1.031, 1.032)
**Ammonium**	1.067 (1.065, 1.068)	1.068 (1.067, 1.070)
**$PM_{2.5}$ mass**	1.009 (1.008, 1.009)	1.009 (1.008, 1.009)

Note:
* Hazard ratios were calculated using the same IQRs as described in Table 1.

Exposure I pollutants were derived from van Donkelaar et al. (2019).
Exposure II pollutants were derived from Amini et al. (2022).
[#]Lag 0: the model was built by linking current year's exposure to current year's health records.
[#]Lag 1: the model was built by linking last year's exposure to current year's health records.

Table S5. Hazard ratios of death per interquartile range (IQR) or per 1 µg/m^3 increase in $PM_{2.5}$ components from single-component models, among the non-mover cohort.

**Pollutants**	**Mortality (N=54,277,906)**	
Per IQR increase	**Exposure I***	**Exposure II***
**Black carbon**	1.008 (1.007, 1.009)	1.054 (1.053, 1.054)
**Organic matter**	1.010 (1.009, 1.011)	1.007 (1.006, 1.008)
**Soil dust**	1.011 (1.010, 1.011)	1.045 (1.043, 1.046)
**Nitrate**	1.027 (1.026, 1.028)	1.026 (1.025, 1.027)
**Sulfate**	1.030 (1.029, 1.031)	1.003 (1.002, 1.004)
**Ammonium**	1.035 (1.034, 1.036)	1.014 (1.013, 1.015)
**$PM_{2.5}$ mass**	1.025 (1.025, 1.026)	1.006 (1.005, 1.007)
Per 1 µg/m^3 increase		
**Black carbon**	1.024 (1.022, 1.026)	1.203 (1.200, 1.207)
**Organic matter**	1.008 (1.008, 1.009)	1.006 (1.005, 1.006)
**Soil dust**	1.028 (1.026, 1.030)	1.137 (1.133, 1.141)
**Nitrate**	1.026 (1.025, 1.026)	1.030 (1.029, 1.031)
**Sulfate**	1.017 (1.017, 1.018)	1.002 (1.001, 1.002)
**Ammonium**	1.042 (1.041, 1.044)	1.018 (1.017, 1.020)
**$PM_{2.5}$ mass**	1.007 (1.007, 1.007)	1.001 (1.001, 1.002)

Note:
*All hazard ratios were calculated using the same IQRs as described in Table 1.
The non-mover cohort for dementia includes a total of 54,277,906 individuals.
Exposure I pollutants were derived from van Donkelaar et al. (2019).
Exposure II pollutants were derived from Amini et al. (2022).

Table S6. Hazard ratios of death per interquartile range (IQR) or per 1 μg/m^3 increase in $PM_{2.5}$ components from single-component models, among male and female subjects.

	Male		Female	
	**Per IQR increase**	**Per 1 μg/m^3 increase**	**Per IQR increase**	**Per 1 μg/m^3 increase**
**Exposure I***				
**Black carbon**	1.019 (1.018, 1.019)	1.057 (1.055, 1.060)	1.019 (1.019, 1.020)	1.059 (1.057, 1.061)
**Organic matter**	1.022 (1.021, 1.023)	1.019 (1.018, 1.019)	1.019 (1.018, 1.019)	1.016 (1.015, 1.016)
**Soil dust**	1.001 (1.000, 1.002)	1.003 (1.000, 1.005)	1.003 (1.002, 1.003)	1.007 (1.004, 1.009)
**Nitrate**	1.056 (1.054, 1.057)	1.032 (1.031, 1.033)	1.070 (1.068, 1.071)	1.040 (1.039, 1.041)
**Sulfate**	1.025 (1.023, 1.026)	1.023 (1.022, 1.024)	1.022 (1.021, 1.023)	1.020 (1.019, 1.021)
**Ammonium**	1.050 (1.048, 1.051)	1.061 (1.059, 1.062)	1.060 (1.058, 1.061)	1.073 (1.071, 1.074)
**$PM_{2.5}$ mass**	1.039 (1.038, 1.041)	1.011 (1.010, 1.011)	1.040 (1.039, 1.041)	1.011 (1.010, 1.011)
**Exposure II***				
**Black carbon**	1.049 (1.048, 1.050)	1.184 (1.179, 1.189)	1.041 (1.040, 1.042)	1.154 (1.150, 1.159)
**Organic matter**	1.029 (1.028, 1.030)	1.025 (1.024, 1.026)	1.024 (1.023, 1.025)	1.021 (1.020, 1.022)
**Soil dust**	1.017 (1.015, 1.018)	1.049 (1.045, 1.054)	1.011 (1.010, 1.013)	1.034 (1.030, 1.038)
**Nitrate**	1.048 (1.046, 1.050)	1.026 (1.025, 1.027)	1.064 (1.062, 1.065)	1.034 (1.033, 1.035)
**Sulfate**	1.031 (1.030, 1.033)	1.035 (1.034, 1.037)	1.028 (1.026, 1.029)	1.031 (1.030, 1.032)
**Ammonium**	1.042 (1.040, 1.043)	1.055 (1.053, 1.057)	1.057 (1.056, 1.058)	1.075 (1.073, 1.077)
**$PM_{2.5}$ mass**	1.034 (1.033, 1.035)	1.008 (1.008, 1.009)	1.035 (1.034, 1.036)	1.009 (1.008, 1.009)

Note:
*All hazard ratios were calculated using the same IQRs as described in Table 1.
Exposure I pollutants were derived from van Donkelaar et al. (2019).
Exposure II pollutants were derived from Amini et al. (2022).

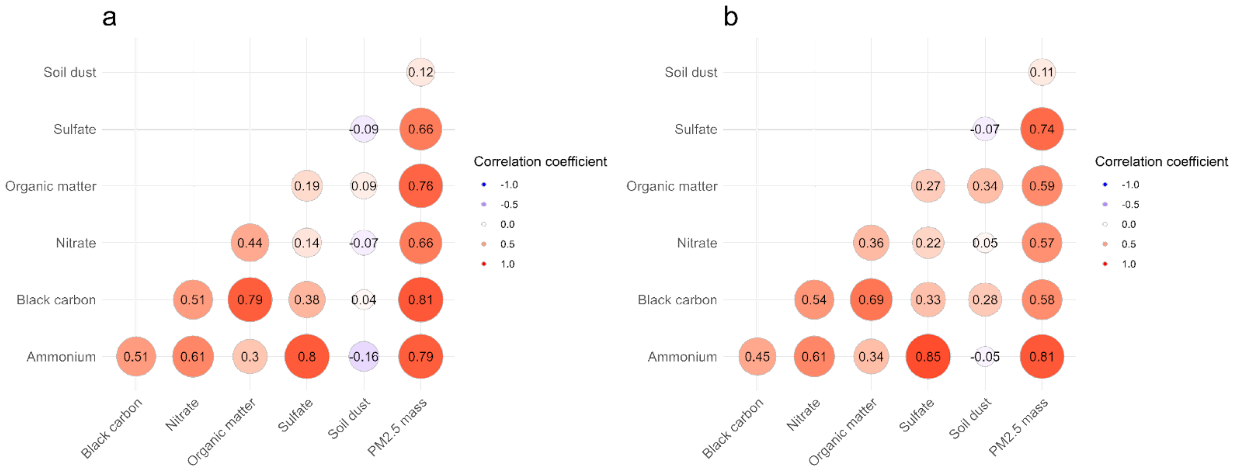

Figure S1. Correlation matrix among PM2.5 mass and its six major components (black carbon, organic matter, soil dust, nitrate, sulfate, and ammonium) for two speciated air pollution datasets (a: Exposure I; b: Exposure II). Exposure I pollutants were derived from van Donkelaar et al. (2019). Exposure II pollutants were derived from Amini et al. (2022).

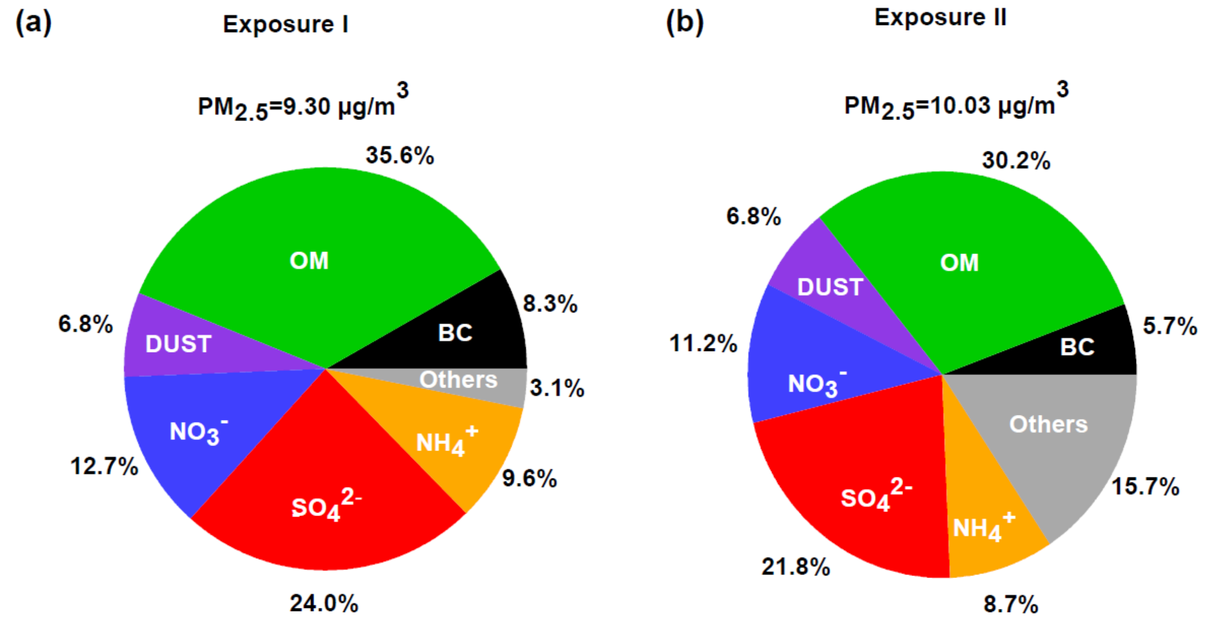

Figure S2. Averaged chemical composition of PM2.5 for two speciated air pollution datasets (a: Exposure I; b: Exposure II) from 2000 to 2017, including black carbon (BC), organic matter (OM), soil dust (DUST), nitrate (NO3-), sulfate (SO42-), ammonium (NH4+), and others. The others are calculated as the difference between the $PM_{2.5}$ mass and the sum of six $PM_{2.5}$ major components of interest. Exposure I pollutants were derived from van Donkelaar et al. (2019), with a $PM_{2.5}$ mass balance constraint (and thus the difference between mass and the sum of six components is relatively small). Exposure II pollutants were derived from Amini et al. (2022), without a $PM_{2.5}$ mass balance constraint (and thus the difference between mass and the sum of six components could be relatively large).
